# Developing a framework for evaluation: a Theory of Change for complex workplace mental health interventions

**DOI:** 10.1186/s12889-023-16092-x

**Published:** 2023-06-17

**Authors:** Fotini Tsantila, Evelien Coppens, Hans De Witte, Kahar Abdulla, Benedikt L. Amann, Ella Arensman, Birgit Aust, Johanna Creswell-Smith, Luigia D’Alessandro, Lars De Winter, Asmae Doukani, Naim Fanaj, Birgit Greiner, Eve Griffin, Caleb Leduc, Margaret Maxwell, Cliodhna O’ Connor, Charlotte Paterson, György Purebl, Hanna Reich, Victoria Ross, Jaap Van Weeghel, Chantal Van Audenhove

**Affiliations:** 1 LUCAS, Centre for Care Research and Consultancy, KU Leuven, 3000 Louvain, Belgium; 2 Research Group Work, Organizational and Personnel Psychology, Faculty of Psychology and Educational Sciences- O2L/WOPP KU Leuven, Louvain, Belgium; 3 Optentia Research Focus Area, North-West University, Vanderbijlpark, South Africa; 4 European Alliance Against Depression, Leipzig, Germany; 5 Centre Fòrum Research Unit, Institute of Neuropsychiatry and Addiction (INAD), Parc de Salut Mar, Hospital del Mar Medical Research Institute (IMIM), Barcelona, CIBERSAM Spain; 6 Univ. Pompeu Fabra, Barcelona, Spain; 7 Department of Psychiatry and Psychotherapy, Klinikum Der Universität München, Munich, Germany; 8 School of Public Health, University College Cork, Cork, Ireland; 9 National Suicide Research Foundation, University College Cork, Cork, Ireland; 10 Australian Institute for Suicide Research and Prevention, School of Applied Psychology, Griffith University, Brisbane, Australia; 11 National Research Centre for the Working Environment, 2100 Copenhagen, DK Denmark; 12 Finnish Institute for Health and Welfare (THL) Equality Unit – Mental Health Team, Helsinki, Finland; 13 International Association for Suicide Prevention (IASP), 5221 Wisconsin Avenue NW, Washington DC, 20015 USA; 14 Phrenos Center of Expertise, Da Costakade 45, 3521 VS Utrecht, the Netherlands; 15 Faculty of Epidemiology and Population Health, London School of Hygiene and Tropical Medicine, Keppel Street, London, UK; 16 Mental Health Center Prizren, Prizren, Kosovo USA; 17 Almae Mater Europaea Campus College Rezonanca, Prishtina, Kosovo USA; 18 Nursing, Midwifery and Allied Health Professions Research Unit, University of Stirling, Stirling, UK; 19 Institute of Behavioral Sciences, Semmelweis University, 1085 Budapest, Hungary; 20 German Depression Foundation, 04109 Leipzig, Germany; 21 Depression Research Centre of the German Depression Foundation, Department of Psychiatry, Psychosomatic Medicine and Psychotherapy, University Hospital, Goethe University, 60528 Frankfurt Am Main, Germany; 22 Australian Institute for Suicide Research and Prevention, School of Applied Psychology, Griffith University, Brisbane, QLD 4122 Australia; 23 Tilburg School of Social and Behavioral Sciences, Tranzo Scientific Center for Care and Welbeing, Tilburg University, Postbus 90153, 5000 LE Tilburg, The Netherlands; 24 Department of Public Health and Primary Care, KU Leuven, 3000 Louvain, Belgium

**Keywords:** Complex interventions, Workplace-based mental health/health interventions, Organizational interventions, MENTUPP, Intervention development, Implementation, Evaluation, Medical Research Council framework, Theory of Change, Small and Medium Enterprises (SMEs)

## Abstract

**Background:**

There is a gap between the necessity of effective mental health interventions in the workplace and the availability of evidence-based information on how to evaluate them. The available evidence outlines that mental health interventions should follow integrated approaches combining multiple components related to different levels of change. However, there is a lack of robust studies on how to evaluate multicomponent workplace interventions which target a variety of outcomes at different levels taking into account the influence of different implementation contexts.

**Method:**

We use the MENTUPP project as a research context to develop a theory-driven approach to facilitate the evaluation of complex mental health interventions in occupational settings and to provide a comprehensive rationale of how these types of interventions are expected to achieve change. We used a participatory approach to develop a ToC involving a large number of the project team representing multiple academic backgrounds exploiting in tandem the knowledge from six systematic reviews and results from a survey among practitioners and academic experts in the field of mental health in SMEs.

**Results:**

The ToC revealed four long-term outcomes that we assume MENTUPP can achieve in the workplace: 1) improved mental wellbeing and reduced burnout, 2) reduced mental illness, 3) reduced mental illness-related stigma, and 4) reduced productivity losses. They are assumed to be reached through six proximate and four intermediate outcomes according to a specific chronological order. The intervention consists of 23 components that were chosen based on specific rationales to achieve change on four levels (employee, team, leader, and organization).

**Conclusions:**

The ToC map provides a theory of how MENTUPP is expected to achieve its anticipated long-term outcomes through intermediate and proximate outcomes assessing alongside contextual factors which will facilitate the testing of hypotheses. Moreover, it allows for a structured approach to informing the future selection of outcomes and related evaluation measures in either subsequent iterations of complex interventions or other similarly structured programs. Hence, the resulting ToC can be employed by future research as an example for the development of a theoretical framework to evaluate complex mental health interventions in the workplace.

**Supplementary Information:**

The online version contains supplementary material available at 10.1186/s12889-023-16092-x.

## Background

Depression and anxiety are the most frequently occurring mental disorders in Europe affecting 21 and 25 million people respectively [1]. Depression often co-occurs with other mental health conditions such as anxiety, stress, and burnout and carries the risk of adverse events in various physical diseases [2–5]. If left untreated, depression is associated with an increased risk of suicidal behavior, another major public health problem to which the European Region has the highest suicide mortality rate at 12.8 per 100.000 population in 2019 [6]. In addition in 2018, 11% of the adult population across European countries reported to having experienced psychological distress [7].

Individuals spend at least one third of their lifetime at work and there is a strong body of evidence showing that prolonged experience of poor mental health due to work-related psychosocial and physical risk factors can exacerbate pre-existing mental illness or result in more severe mental health symptoms [8]. Correspondingly, poor mental health is associated with fear of stigmatization and lower stress-tolerance preventing people from having a positive work-life experience. Mental illnesses and their treatment entail high individual and societal consequences. Based on the Health at a Glance Europe 2018 report, the total costs of mental health problems are estimated to be higher than 4% of GDP (more than €600 billion) across the counties of the European region [9]. According to the European Agency of Safety and Health at Work, the total cost of mental disorders in Europe is estimated at €240 billion per year. This amount consists of €97 billion per year for direct healthcare costs such as prevention and treatment, €9 billion per year for non-medical costs like social services, and €133 billion per year for indirect costs including absenteeism and presenteeism [10, 11].

Literature has shown that mental health promotion interventions in the workplace have been strongly associated with reduced absenteeism, presenteeism, and increased productivity [12, 13]. However, there appears to be a lack of workplace-based interventions which promote mental health, particularly in the context of Small and Medium Enterprises (SMEs) [14]. Even though SMEs are the backbone of Europe’s economy [15], few studies on the implementation of actions that promote mental health involving SMEs have been reported [14, 16]. Reasons behind this may relate to limited capacity and financial resources, lack of awareness and competencies to adopt and integrate such programs, or lack of interest [17]. Mental health promotion is an often neglected area in the workplace, further exacerbated by challenges concerning the COVID-19 pandemic [18, 19]. More research in terms of the development, implementation, and evaluation of interventions that promote mental health at work is therefore especially needed to focus on SMEs.

Existing frameworks for workplace mental health describe a broad approach which helps employees stay mentally healthy at work, recognise early signs of mental health problems, assist them in finding professional treatment and provide support when returning to work following recovery [20, 21]. Recent research has shown that integrated approaches to mental health promotion are desirable for the development and implementation of interventions in the workplace, as they tend to take a more universal approach to address a wide range of the different needs of the working population and do not limit themselves to only one or a few individual outcomes [20]. LaMontagne and colleagues [21] developed a framework integrating the importance of the protection of mental health by reducing work-related risk factors and the promotion of mental health in the workplace by developing the positive aspects within the workplace, and addressing the need to manage mental illness as necessary among working populations. A more recent, evidence-based theoretical framework by Petrie and colleagues [20] indicates that an integrated mental health intervention implemented in occupational settings should combine five main strategies. Firstly, the intervention should be aimed at minimizing harm. Secondly, management should focus on the enhancement of organizational resilience. Thirdly, personal resilience should be enhanced through the promotion of mental and physical health. Fourthly, help-seeking behavior should be facilitated, and fifthly, recovery and return to work after sick leave should be promoted.

These interventions can also be considered to take a multilevel approach as they target the micro-social level of individuals and their health capital as well as the organizational level of their workplace [22]. Integrated interventions can also employ multiple strategies and mechanisms to achieve a variety of outcomes at the same or different levels. Although multilevel interventions seem to be appropriate to address the complexity of mental health at work, it remains a challenge to evaluate these types of interventions. Furthermore, scholars have criticized organizational intervention research for failing to incorporate the multiple levels of the design of interventions in their evaluation strategy [23, 24].

Mental Health Promotion and Intervention in Occupational Settings (MENTUPP) is a Horizon 2020 funded project which aims to improve mental health in the workplace by developing a complex evidence-based multilevel intervention inspired by the integrated approach of Petrie and colleagues [20]. The intervention targets both non-clinical (stress, burnout, wellbeing, depressive symptoms) and clinical (depressive and anxiety disorders) mental health symptoms, as well as combating stigmatizing attitudes. The project specifically focuses on SMEs within the construction, healthcare, and information and communication technology (ICT) sectors. These sectors have been selected as they have been linked to high levels of stress and negative mental health outcomes [25, 26]. The intervention will be implemented and evaluated in nine different countries (Albania, Australia, Finland, Germany, Hungary, Ireland, Kosovo, the Netherlands, and Spain), first in a 6-month pilot study and then in a large cluster randomized controlled trial (cRCT) [13]. The MENTUPP intervention is a representative example of a complex intervention not only because it consists of multiple interacting components and tools, but also because it targets different audiences: employees with different job profiles (micro-social level), who work in different organizations and sectors (intermediate level), from different country contexts (macro social level). The components of the MENTUPP intervention are delivered online via the MENTUPP Hub platform, where the intervention components (see "Intervention components" section) are made available for employees and their supervisors.

### Aim

The article aims to describe a program theory that was developed within the context of the MENTUPP project to facilitate the evaluation of its pilot study and further inform a clustered randomized control trial (cRCT) that will follow. The resulting program theory will be beneficial in many ways. During the planning of the evaluation, it helps to select the most appropriate/relevant outcomes and thus supports those relevant results that are available to investigate the evidence. After the intervention, the program theory will help to identify what aspects of the intervention worked as expected and which worked differently, maybe dependent on different circumstances. This knowledge can facilitate the transferability of the intervention across different settings. Finally, the program theory will help to systematically assess how the intervention worked. A profound understanding of the mechanisms that make the intervention work is crucial to convince decision-makers to implement the intervention more widely [27].

## Methods

### Study design

This study uses Theory of Change (ToC) and follows a participatory approach to its development process. ToC brings together the insights from the interdisciplinary members of the MENTUPP consortium collected during workshops. These insights are combined with the findings of six systematic reviews and an expert consultation survey which was distributed to 146 external experts across the nine countries involved in MENTUPP. The systematic reviews and the expert consultation were conducted by the MENTUPP consortium and some of the findings have been already published [14, 28] whereas the rest of them will be published elsewhere in the future.

### The Theory of Change approach and terminology

The developed program theory is based on the guidance provided to researchers by the updated Medical Research Council (MRC) framework [27] to select and apply appropriate methods to implement and evaluate complex interventions. According to the MRC framework, a program theory is needed to describe how, and why an intervention is expected to generate a particular effect, identifying which components of the intervention are most influential and under what circumstances. Moreover, attention should be given to the contextual factors and conditions that are needed to realize the effect. The development of such a theory should involve the consultation of stakeholders to achieve a deeper understanding of the mechanisms within the program, and of the key uncertainties that should be discovered by the program.

There is a variety of reasonings behind the selection of the Theory of Change (ToC) as the most appropriate program theory approach to evaluate the complex MENTUPP intervention. As described by Breuer and colleagues, ToC is an approach that can help explain the modus operandi of a complex intervention [29]. ToC formulates assumptions that are associated with the different intervention components and makes explicit the way in which particular long-term outcomes can be generated through a logical sequence of intermediate outcomes. Creating a ToC can provide a detailed description of the reasons why the desired change is expected to occur in a specific context and via a certain program. It intends to map out or fill in the “missing middle” between the factors that can bring about changes through a program (its activities or interventions) and how these changes are linked to the targeted outcomes [29]. Furthermore, the opportunity to understand the multifaceted adoption which is required when interventions are delivered at different levels and to different target groups is provided. ToC can shed light on the diversity in implementation between settings, groups, and representativeness of people that are involved.

To develop a ToC, backward mapping is used. The approach starts with identifying the expected long-term objectives of an intervention and then works backward to identify the intermediate outcomes and the causal pathways that are assumed to be required for the long-term objectives to occur [30]. In addition, the components of the intervention are connected to the outcomes to further explain how change happens within the context of MENTUPP. The underlying rationale behind the choice of the intervention components and the selection of outcomes is specified. In the end, a ToC map that graphically displays the whole mechanism of change is constructed.

ToC uses common terminology and shared definitions, which are presented in Table 1 to describe the mechanism of change of an intervention [30]. In addition to the terminology introduced by De Silva and colleagues [30] and analogous to the categorization of outcomes in the model of Fridrich and colleagues [31], in this ToC, we make a distinction between three types of outcomes: long-term, intermediate and proximate outcomes (see Table 1 for definitions).

**Table 1:** The MENTUPP ToC terminology adapted from De Silva et al., 2014, [30] and Fridrich et al., 2015 [31]

Terminology	Definitions
Impact	Real-world change (ultimate goal) that the project is trying to achieve (not measured in the project)
Ceiling of accountability	The point at which implementers/researchers stop measuring whether outcomes have been achieved
Long-term/distal outcomes	The measurable outcomes that the program can achieve on its own. This can inspire the selection of primary and secondary outcome indicators for the evaluation
Intermediate outcomes	Necessary stepping stones (conditions, requirements, elements) that need to be realized for the desired long-term outcomes to be achieved
Proximate outcomes	Results of the change process that are assumed to immediately arise
Intervention components	Certain activities or strategies that need to be undertaken to bring about outcomes
Assumptions	External conditions beyond the control of the project that we assume are in place so the intervention functions successfully and intermediate outcomes are achieved
Rationales	The facts or reasons (based on evidence or experience) behind the choice of the intervention components or the selection of outcomes that justify the assumed causal pathway
Indicators	Operationalization of outcomes to measure whether they are reached

### Development steps of the ToC

Four steps were undertaken to develop the MENTUPP ToC: 1) the synthesis of the evidence obtained from the six systematic reviews and the expert consultation. This approach was used to identify available interventions, as well as the barriers and facilitators of the implementation of mental health interventions in the workplace [13]. 2) An introductory meeting with members of the MENTUPP consortium was conducted to introduce and harmonize the ToC approach. 3) Four workshops were held with the MENTUPP consortium to present a suggested ToC and further develop it. Finally, 4) a series of internal meetings with the core research team were held to refine and finalize the ToC. The following section further describes the participants involved in the ToC workshops and the procedure followed to develop and refine the MENTUPP ToC.

### Participants in ToC workshops

In this study, all members of the MENTUPP consortium were invited by e-mail to participate in the development of the ToC. The consortium members contributed to the development and implementation of the intervention, so they had a profound understanding of the intervention. Of the 54 consortium members, 24 actively participated in at least one meeting or workshop (see Table 2 for more details)*.* The participants consisted of academics representing 11 European countries with expertise in mental health and wellbeing, suicide prevention, mental health workplace intervention research, occupational mental health disorders, stigma in the workplace, implementation science, health economics, and biostatistics among other relevant expertise.

### Procedure

The four steps we undertook to develop the ToC are elaborated further through an overview of the purpose, method, and output of six process stages that were followed and are presented in Table 2.

During step 1, the findings deriving from the six systematic reviews and the expert consultation were synthesized and in conjuction with additional scientific knowledge and theories they were exploited to design a first draft of the MENTUPP ToC. This preliminary ToC which integrated the evidence underlying the development and implementation of the intervention was presented to the first ToC workshop as an inspirational example of the way the MENTUPP ToC components could be selected. An overview of the most significant rationales (see Table 1 for defintion) that were used to explain the selection of the outcomes, the way in which outcomes are connected to each other, and the way in which certain activities and interventions are believed to lead to particular outcomes can be found in the supplementary materials accompanying this article (see Table S1).

While ToC has been used frequently in a research context, it requires specific knowledge and skills from all participants [30]. Hence, the trajectory of participant consultations started with an introductory meeting (step 2) in which the terminology, the definitions of the key elements, and the procedure used were explained. The introductory meeting was followed by four workshops (step 3), aiming to gradually reach a consensus on the different parts of the MENTUPP ToC. Each workshop started with a short introduction about ToC to refresh everyone's knowledge, a presentation of the elements of the MENTUPP ToC that had been revised based on the input of the preceding workshop, and an overview of the main objectives of the group discussion that would follow. To prepare participants for the workshops, an outline of the latest version of the MENTUPP ToC and the main questions that would be the focus of the upcoming workshop was distributed via e-mail beforehand.

Group discussions were moderated by one of the members of the core research team researchers and audio and video recorded (following verbal consent) in order to allow for accurate processing. The purpose, method, and output of the workshops is elaborated further in Table 2. After each workshop, participants were invited to e-mail additional comments on the workshop or the ToC to the core research team members who were also responsible for the evaluation part of MENTUPP. In between workshops, comments and suggestions were discussed by the core research team, and the MENTUPP ToC was iteratively revised before finalization (step 4).

**Table 2:** Purpose and output of the MENTUPP ToC process stages

Process stages	Purpose	Output
1. Exploitation of the evidence base collected through multiple reviews and an expert consultation	The knowledge derived was utilized to define the MENTUPP ToC outcomes	Through the reviews findings on: 1) mental health interventions in the construction industry, 2) mental health interventions in the ICT sector and 3) mental health interventions in the health sector, 4) interventions to detect and treat depression and anxiety in SMEs, 5) interventions to reduce stigma towards mental illness in the workplace, and 6) the implementation of mental health interventions in the workplace were collected Through the expert consultation findings on the insights and assessments of stakeholders from 8 European countries and Australia on: 1) the promotion of employee wellbeing, 2) the mental health needs of employees and employers in addition to identifying any gaps, 3) the level of stigma and gaps in anti-stigma programs in the workplace, 4) gender-specific needs, 5) the impact of the COVID-19 pandemic, and acceptability of interventions were collected. All the findings were merged and thematically analyzed and synthesized resulting to:1) a list of outcomes which MENTUPP aims to achieve, 2) a list of intervention components that are required for the outcomes to be achieved, 3) formulation of recommendations concerning implementation
2.Introductory ToC meeting March 1st 2021 (*n* = 9)	To introduce ToC, to achieve consensus on its selection as an appropriate theoretical framework for the development approach. To discuss a first proposal of key components	Through this online meeting we achieved: 1) the approval of the ToC and development approach, 2) a deeper understanding of its terms and defitnions, and 3) the exchange of ideas about MENTUPP key components (impact, ceiling of accountability, long-term and intermediate outcomes, rationales, assumptions)
3.First ToC workshop March 23rd 2021 (*n* = 24)	To further familiarize with the ToC approach and discuss a second proposal on the MENTUPP key components	Through this online workshop we achieved: 1) consensus on impact, ceiling of accountability and long-term outcomes, and 2) the further elaboration of the intervention components and their expected outcomes by the developers
4.Second ToC workshop May 20th 2021 (*n* = 20)	To create a first draft of the MENTUPP ToC map	Through this online workshop we achieved: 1) the classification of the intervention components and the outcomes in broader thematic clusters to reflect better the expected level of change, 2) the link between the outcomes and three levels of change: a) the leader, b) the employee and c) the SME level, 3) the optimization of the rationales and the assumptions, and 4) the reformulation of the ToC map
5.Third ToC workshop July 1st 2021 (*n* = 19)	To discuss the second draft of the ToC map including the expected causal pathways, the clustering of the intervention components and a first proposal of indicators	Through this online workshop we achieved: 1) the categorization of the outcomes in long-term/distal, intermediate and proximate highlighting the causal mechanisms within MENTUPP, 2) the link between the outcomes to a fourth level of change: the team level, and 3) the inclusion of outcomes related to implementation requirements in the ToC map, 4) the selection of outcome indicators to describe the main changes expected to occur due to MENTUPP, and 5) a first proposal of assumptions indicators
6.Fourth ToC workshop September 24th 2021 (*n* = 17)	To discuss and finalize the third draft of the ToC map including expected backwards interactions. To discuss the second proposal of the outcomes indicators. To discuss the assumptions indicators	Through this online workshop we achieved: 1) the finalization of the ToC map and the ToC key components, 2) the finalization of the long-term, proximate, intermediate outcomes indicators as well as of the assumptions indicators, and 3) the writing of the ToC narrative

### Data – analysis

The audio and video recordings of the group discussions of the four workshops were transcribed verbatim. A report was produced summarizing all comments prior to all recordings being then destroyed due to privacy considerations. Based on the received input, the ToC was systematically reworked and the assumed causal mechanisms between the selected outcomes were illustrated in a ToC map which was constructed using the Lucidchart software (http://www.lucidchart.com), which helps its users sketch and share flowchart diagrams.

## Results

**Fig. 1:**
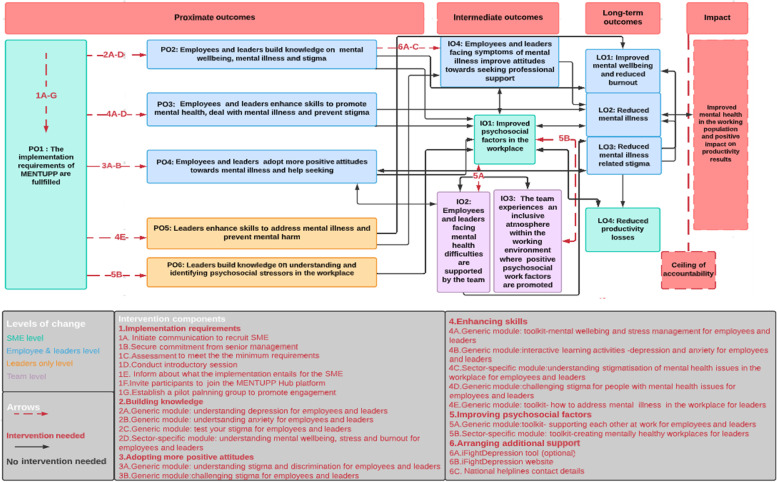
The MENTUPP ToC map

The final version of the MENTUPP ToC that was developed consists of seven key components, including: 1) proximate outcomes, 2) intermediate outcomes, 3) long-term outcomes, 4) ceiling of accountability, 5) impact, 6) intervention components, and 7) assumptions. The first six components are graphically presented in the ToC map (see Fig. 1) and the seventh is elaborated further in the "Assumptions" section.

In general, the arrows between the boxes represent the way one outcome is expected to lead to another and finally to the long-term outcomes. However, ToC goes beyond linearity including bidirectional and circular causal pathways between the outcomes as indicated by the double-pointed arrows in the ToC map.

Furthermore, the red arrows represent links between outcomes that are expected to occur directly when certain intervention components are applied, whereas the black arrows show the expected indirect effects of the intervention. Each intervention component is connected to a different outcome on the map.

### Proximate and intermediate outcomes

The achievement of the intermediate outcomes must precede the long-term outcomes, so we expect that they will be achieved on an earlier stage (during the second and the fourth month), whereas the long-term outcomes are expected to be visible after the six-month MENTUPP implementation. As it concerns the proximate outcomes, they are expected to occur during the first months of the implementation period. The proximate and the intermediate outcomes are expected to occur at four levels: 1) at the individual level of all employees at all grades in the participating SMEs including leaders, as individuals who also are affected by their work environment and who also can have mental health problems, 2) at the group or team level of the interactive peers, 3) at the leader level referring to the role of supervisors and managers and their responsibility for the mental health of their employees, and 4) at the organizational level. This division corresponds to the four levels of: Individuals, Groups, Leaders, and Organization (IGLO) of the Context-Process-Outcomes (CPO) evaluation model of Fridrich and colleagues [31]. Based on the results of the reviews, the expert consultation, and the four ToC workshops, we identified six proximate and four intermediate outcomes (POs and IOs) which are located on the left side of the map (Fig. 1).

The first proximate outcome that we identified is “the implementation requirements of MENTUPP are fulfilled” (PO1). We expect that this outcome will occur at the organizational level of the SME and is necessary for the other five proximate outcomes to occur. Moreover, we foresee that this outcome will be achieved via activities from the project group to introduce the intervention to future participants and to facilitate engagement during the implementation of the intervention [13].

The next proximate outcome is “employees and leaders build knowledge on mental wellbeing, mental illness and stigma” (PO2). Enhanced knowledge can promote a person's actual ability to perform a behavior. According to the Social Cognitive Theory developed by Bandura [32], health promotion and illness prevention can be achieved by teaching health promotional actions. Moreover, in the context of mental health, there is evidence indicating that enhancing mental health literacy, defined as “increasing knowledge and beliefs about mental disorders which aid their recognition, management or prevention” [33] is closely related to taking action to promote one’s own mental health or that of others [34]. There is also extensive literature to show that interventions exploiting CBT-based and/or psychoeducational materials are able to reduce symptoms of depression and anxiety [23, 35–37]. More specifically, Martin and colleagues [23] managed to decrease psychological distress of SME managers using psychoeducational and CBT-based materials after implementing their intervention for four months. Saraf and colleagues [38] achieved an improvement on depressive and anxiety symptoms of SME entrepreneurs through their three-month intervention. Moreover, the CBT-based intervention of Sorensen and colleagues [35] which was applied in Danish workplaces achieved significant positive changes in psychological distress symptoms and symptoms of anxiety over a period of 12 months.

The third proximate outcome (PO3) is “employees and leaders enhance skills to promote mental health, deal with mental illness and prevent stigma”. This outcome is supported by the Theory of Planned Behavior which considers skills as a motivational factor for one’s perception of control over a behavior. This means that when a person intends to perform a behavior (e.g. pay more attention to one’s own wellbeing), enhanced skills will help the person to successfully achieve it [39].

“Employees and leaders adopt more positive attitudes towards mental illness and help-seeking” is the fourth proximate outcome we identified (PO4). Stigmatizing attitudes towards mental illness reduces help-seeking and are a major barrier to receiving treatment [40, 41]. Undoubtedly, adopting supportive attitudes toward the potential for treating mental illness should be a key factor in mental health interventions, enabling people to express mental health difficulties and search for appropriate support when needed.

Assuming that the implementation requirements are fulfilled (PO1), then the enhancement of knowledge and skills and the adoption of more positive attitudes towards mental illness (PO2, PO3, PO4) are assumed to be achievable. We consider that the proximate outcomes within the blue boxes in Fig. 1 to be fundamental, in that they have to be achieved at the individual level by employees of all grades. The proximate outcomes are assumed to lead to long-term outcomes, and they can contribute to the improvement of psychosocial factors in the workplace(IO1). The intervention is available on a universal basis in the workplace i.e., the individual level should include all employees in the organization following the assumption that even people who do not suffer from mental illness are expected to benefit from mental health promotion in the workplace [21, 42]. It also means that all individual employees at all levels can benefit from mental health promotion in the workplace as they all have personal mental health needs [43].

The proximate outcomes at the leader level are illustrated by two yellow-colored boxes in Fig. 1 (PO5, PO6). These outcomes describe the changes that are expected from the leaders with regard to their role as leaders and refer to their skills and activities in relation to employees’ mental health in the workplace.

The fifth proximate outcome expected at the level of the leaders is that “leaders enhance skills to address mental illness and prevent mental harm” (PO5). Addressing employees’ mental health needs in the workplace context is considered to be a crucial component of a genuinely integrated approach [21]. Psychoeducation for people in a leading role within an organization may provide increased understanding and practical solutions to offer to employees [21].

The final proximate outcome we identified is that “leaders build knowledge on understanding and identifying psychosocial stressors in the workplace” (PO6). There is evidence to suggest that mental wellbeing can be promoted by enhancing positive aspects of work and reducing work-related risk factors [21, 36]. To this end, leaders need to increase their knowledge and skills as they are key persons to implement change at the organizational level.

Column 3 of the boxes on the map in Fig. 1 depicts the intermediate outcomes. All proximate outcomes that were described above are perceived as key elements to achieve “improved psychosocial factors in the workplace” (IO1). We expect this outcome at the organizational level (green color), but it will be achieved through proximate outcomes at the individual level of all employees (blue color), at the level of leaders (yellow color), and through the interaction between peers (purple color). The psychosocial factors that are targeted through the MENTUPP intervention and that are linked to this outcome, are the influence that employees have on their work, the quality of leadership they experience, the social support that they receive from colleagues and supervisors, the existence of mutual trust between employees and of trust regarding the management and the justice experienced in the workplace.

The intermediate outcomes also include two assumed outcomes at group or team level (purple boxes). One of the intermediate outcomes on the map expected to occur at the team level is that “employees and leaders facing mental health difficulties (meaning mental health distress and/or illness) are supported by the team” (IO2). The assumption here is that the promotion of self-organized peer support in the workplace is believed to help prevent and deal with mental health difficulties through the promotion of help-seeking and providing help behaviors [44].

The next intermediate outcome (IO3) which we expect to derive from the team level is that “the team experiences an inclusive atmosphere within the working environment where positive psychosocial factors are promoted”. This outcome is based on the assumptions that peers have to (a) experience that changes occurred at the organizational and the team level, and (b) perceive these changes as helpful (c)in order to disclose their mental health difficulties and receive support within the working environment [45, 46]. IO2 and IO3 are expected to derive from and add to “improved psychosocial factors in the workplace” (IO1) and are also linked to the long-term outcomes of the intervention.

The last intermediate outcome we expect is “employees and leaders facing symptoms of mental illness improve attitudes towards seeking professional support” (IO4). This outcome is linked to the proximate outcome of building knowledge (PO2). This outcome is based on the assumption that if people experiencing mental health difficulties are able to recognize a need for support and know where to find it, they will be more likely to seek professional help. All intermediate outcomes (IO1—IO4) are expected to lead to the long-term outcomes.

### Long-term outcomes

We expect that four long-term outcomes (LOs) will arise from the MENTUPP intervention in the workplace: (LO1) “improved mental wellbeing and reduced burnout”, (LO2) “reduced mental illness”, (LO3) “reduced mental illness related stigma”, and (LO4) “reduced productivity losses”. Even though the long-term outcomes are expected to occur after the six months, we speculate that outcomes on productivity losses may need more time as they are dependent to the mental health-related long-term outcomes of our intervention.

The long-term outcomes LO1, LO2, LO3, are expected to affect all employees at the individual level (indicated by blue colored boxes in Fig. 1), whereas the fourth long-term outcome LO4 relates to the organizational level (green color). We assume that the relationship between the long-term outcomes and the improvement of psychosocial factors (IO1) is bidirectional as there can be mutual benefits.

Finally, the long-term outcomes (LO1-LO3) are linked to the achievement of the fourth long-term outcome (LO4) concerning the reduction of productivity losses which is a desired change located at the organizational level.

### Impact

The real-world change that is endeavoured through the long-term outcomes of the MENTUPP intervention is defined as “improved mental health in the working population and positive impact on productivity results”.

### Ceiling of accountability

The ceiling of accountability is located between the impact of MENTUPP and its long-term outcomes. This indicates that MENTUPP can be credited for promoting mental health in the workplace, although it cannot account for factors that lie outside the project’s sphere of influence. Hence, this is the threshold beyond which the outcomes of the intervention will not be measured anymore by the researchers.

### Intervention components

The MENTUPP intervention consists of 23 intervention components embedded within an online platform designed to achieve the proximate, intermediate, and long-term outcomes. The 23 components relate to six overarching domains. They can be further divided into seven activities in domain 1 that need to take place within the organization and 16 informative and psychoeducational components in domains 2–6 that are embedded in the online platform, so that they can be used by the participants of the intervention.

#### Domain 1: Implementation requirements

This domain includes seven activities that the project group is planning to conduct to assure that the intervention is initiated appropriately. The first activity involves communicating with the SME leaders and successful recruitment to the MENTUPP research project. The second activity requires commitment from management and willingness from their side to support and promote the intervention. The third activity is to conduct a pre-implementation assessment establishing a project planning group to promote implementation, outlining data confidentiality, and ensuring participants can engage with the intervention during paid working hours. The fourth activity is to conduct an introductory session with the leaders of the SME to introduce them to the informed consent, the purpose of the intervention, the evaluation measures, and the focus groups. The fifth activity is to inform the SME employees about participation and how it can be achieved through their access to the MENTUPP Hub. The sixth activity is an invitation to the participants to access the Hub. The seventh activity involves establishing a planning group including workplace management in the implementation process and facilitating engagement.

#### Domain 2: Building knowledge

Four psychoeducational components are sought to build participants’ knowledge base about mental health in the workplace. The first and the second components focus on a better understanding of depression and anxiety, their impact on work, and treatment options. The third component provides a test to assess attitudes and behavioral intentions toward people with mental illness. The fourth component in this domain is to better understand mental wellbeing, stress, and burnout emphasizing what everyone in the organization can do to support mental health and wellbeing.

#### Domain 3: Enhancing skills

Five psychoeducational components representing this domain aim to enhance the mental health skills of employers and employees. The first component in this domain is targeted at laying the knowledge foundations for understanding how mental health and wellbeing at work can be strengthened by everybody in the organization providing practical exercises based on a Cognitive Behavioral Therapy (CBT) approach to deal with unhelpful thoughts, introducing emotions arising under stress, and presenting practical exercises for managing stress, such as breathing and mindfulness techniques. The second component teaches participants to identify symptoms of depression and anxiety, as well as to develop help-seeking skills. Employees can use the third component to better understand how stigmatization associated with mental illness can be expressed in their work sector and adopt sector-specific coping strategies that are provided. The fourth component serves to learn how to react when being stigmatized and to build communicational skills to properly talk about mental health problems. The fifth component only applies to leaders of the organization and supports their understanding of the business impact of depression and anxiety, providing guidance on how to talk about mental health and support to employees who they suspect are depressed, anxious, have suicidal thoughts, or those who are returning to work having been on mental health-related sick leave.

#### Domain 4: Adopting more positive attitudes

There are two components in this domain, the first aims to improve participants’ understanding of mental illness-related stigma and its connection to social stereotypes and the second relates to recommendations for how to reduce stigma at work and how to improve communication about mental health.

#### Domain 5: Improving psychosocial factors in the workplace

The intervention components that are categorized in domains 2, 3, and 4 are related to outcomes at the individual level of all the employees and of the leaders only. However, two further practically oriented toolbox components in this domain are connected to outcomes at the organizational and team level. The first component aims to deepen the understanding of peer support and establish a culture of supporting each other at work. The second component is designed to help leaders achieve a better understanding of mental wellbeing, stress, and burnout in the context of the workplace. It also includes the identification of psychosocial work environment factors that may influence mental wellbeing, stress, and burnout. Furthermore, it includes suggestions to improve communication with staff about psychosocial work environment factors and to address these factors engaging employees in the development of these processes and initiation of related action plans.

#### Domain 6: Arranging additional support

It is anticipated that some of the participants will discover a need for additional support to overcome mental illness. To address this possibility and help prevent suicidal behavior, the iFightDepression tool—an internet-based self-management program for people with milder forms of depression (https://ifightdepression.com/en/self-management-resources/ifightdepression-tool) was introduced in some partner countries if it became apparent that a participant may is in need of additional support. However, this component is not available to all the implementation countries via MENTUPP although it is partially available for general use via the iFightDepression website. Through this online platform key information about depression, self-help resources, and contact details of help services are provided. Additionally, participants are provided with a third component which is the contact details of the national mental health helplines of the countries involved in the intervention. The intervention components arranging additional support for employees and leaders have been designed to support them during the implementation period if needed, but they can also be exploited as resources of additional help in the long-term.

### Assumptions

The outcomes of the MENTUPP ToC can be influenced by the intervention components, but also by the assumptions outlined in Table 3 below. Whereas the intervention components are part of the intervention and thus, can be managed within the context of the project, assumptions are not part of the intervention and lie beyond the control of the project. Nevertheless, assumptions need to be met for the outcomes to occur.

The assumptions in a ToC are to some degree comparable to the omnibus context that is referred to by Fridrich and colleagues [31]. The omnibus context refers to aspects related to the general intervention and the implementation setting and are hardly or not at all manipulable by implementers (e.g. economic situation), but may have an influence on the implementation [31]. Based on consultation with the participants of the ToC workshops, we identified ten assumptions that need to be true for our results to be achieved which are presented in Table 3.

**Table 3:** Overview of the MENTUPP ToC assumptions

Assumptions	Reasoning
1. The national culture is in favor of promoting mental wellbeing and talking about mental illness	Research has shown that the national culture of a country may have an impact on the beliefs, attitudes, coping strategies, and help-seeking behaviors of citizens [47]. We assume that a general attitude about mental health in a country will probably have an influence on how the MENTUPP intervention is used and that countries in which there is a more open attitude about mental illness will promote the acceptability of the intervention
2. The SME has sufficient resources	We assume that the participating SMEs have sufficient resources (time, financial resources, human capacity) all through the implementation period.Even if the availability of resources is discussed beforehand with the director of the SME during recruitment, SMEs often struggle with new financial constraints in order to survive and grow
3. The SME has an organizational culture that supports MENTUPP	We assume that SMEs who choose to participate in MENTUPP are to a certain degree interested in mental health promotion and preventing mental illness in employees and perceive it as useful
4. The SME has a current need for mental health promotion and prevention of mental illness	We assume that the MENTUPP intervention will be appreciated particularly by SMEs that strive to promote employees’ mental wellbeing and support employees with mental illness
5. Employees have internet access	Without internet access employees cannot make use of the internet-based MENTUPP intervention. We, therefore, assume that employees have access to the internet, a computer, and/or a mobile phone
6. Employees have proficiency in the language that is used by MENTUPP	The development of the intervention components takes into consideration that the language used has to be tailored to the language level of the participants. However, we assume that there is a possibility that not all the needs are met with respect to language proficiency
7. MENTUPP fits into the daily routine of employees	We assume that participants will use the MENTUPP Hub if it fits into their daily routines. It is recommended that, the MENTUPP Hub is mostly used during working hours. However, people occupied in SMEs often face lack of time due to multiple challenges that they have to handle
8. New employees are actively involved in the intervention	For MENTUPP to become sustainable, the SMEs have to systematically introduce new employees to the MENTUPP intervention and provide them access to the Hub
9. Implementation is disrupted as little as possible by unexpected events on a national or organizational level	We assume that the implementation of MENTUPP has the potential to be adapted to unexpected events and public health emergencies at national level (e.g., the COVID pandemic, a natural disaster, the loss of an influential person) or on the organizational level (e.g., a sudden change in management, the dismissal of a key employee, an impending bankruptcy)
10. MENTUPP is supported by mental health professionals independent from the project	For MENTUPP to be more effective, the existence of external mental health professionals supporting the intervention and providing the participants with additional help is required. We assume that the provision to refer to additional tailored mental health services and support as required will be promoted
11. Leaders’ decision making related to working environment is influenced by MENTUPP	We assume that MENTUPP will inspire leaders to follow work processes, policies, and structures in favor of mental health promotion in the workplace

### Indicators

An important advantage of Theory of Change is that it improves the evaluation of complex interventions by identifying meaningful evaluation indicators linking them to the expected long-term, intermediate and proximate outcomes. The evaluation strategy of the pilot study relies on a comprehensive mixed method design which consists of a combination of quantitative and qualitative measures [13] which were connected to the indicators identified by the ToC. This allows us to examine whether MENTUPP generates the expected outcomes as prescribed in the ToC and to test more specific hypotheses.

Furthermore, the developed program theory will be used in conjunction with a comprehensive process evaluation including the ToC assumptions (Sect. 3.6). The diversity between the involved countries, work sectors, size of enterprises and participant characteristics is expected to have an impact on implementation. Therefore, indicators have also been developed to assess the ToC assumptions. This way, contextual factors external to the intervention and their impact on the outcomes and the implementation can be evaluated. The differences between intervention contexts will be used as moderators to indicate barriers and facilitators to implementation.

We will thoroughly report on the MENTUPP outcome and process strategy and results in upcoming publications, but we shortly present here an example of how the indicators developed through the ToC will facilitate the evaluation of this complex intervention.

The ToC map describes the assumed associative relationship between PO4 (employees and leaders adopt more positive attitudes towards mental illness and help seeking) and IO2 (employees and leaders facing mental health difficulties are supported by the team) which in turn causes LO3 (reduced mental illness related stigma). In order to examine this relationship, we have developed three indicators which can be measured with validated scales: 1) attitudes towards mental illness and help seeking, 2) social support by colleagues, and 3) personal stigma towards mental illness respectively. Then, linear mixed models can be used to take two levels of clustering in the data into account. The two levels would be employees and leaders within the participating SMEs. Baseline and post-intervention data will be used to identify the differences in: 1) attitudes towards mental illness and help seeking, 2) social support by colleagues, and 3) personal stigma towards mental illness between the groups. The causal relationship between proximate, intermediate, and long-term outcomes will be examined using regression analysis techniques.

## Discussion

Based on recommendations from the updated MRC framework, we developed a ToC to guide the MENTUPP research project and inform the development, implementation, and evaluation of complex mental health interventions in the workplace. As a result of the developed ToC, the procedure through which the ultimate goal of the intervention is assumed to be pursued is made explicit and visualized in a structured and logical ToC map. The ToC allowed us to identify multiple proximate and intermediate outcomes, beyond a single primary outcome, which would not capture the potential impacts of such a complex intervention. In this regard, the ToC explains how the hypothesized causal pathways pursued through different levels of change were initiated by the multiple intervention components. The ToC map in Fig. 1 explains trajectories we assume will be followed to accomplish the long-term outcomes of the MENTUPP intervention in the workplace: LO1) “improved mental wellbeing and reduced burnout”, LO2) “reduced mental illness”, LO3) “reduced mental illness-related stigma”, and LO4) “reduced productivity losses”. Through the achievement of the MENTUPP long-term outcomes, we aim to improve mental health in the working population and as result to achieve positive impact on productivity (referred to as the MENTUPP impact).

The main purpose of developing a ToC for the MENTUPP project was to provide a comprehensive theory of how complex mental health interventions in the workplace are expected to achieve change as well as to provide a framework for their evaluation. Following the checklist provided by Breuer and colleagues [29], we used the resultant ToC to develop the following evaluation research questions: (a) is the intervention effective? (b) does the intervention generate the expected long-term, intermediate and proximate outcomes as prescribed in the ToC? (c) what would be an appropriate design of a process evaluation to accompany the ToC? (d) how can we assess the role of the context using the framework and what are the most important characteristics to take into consideration when evaluating a complex mental health intervention in the workplace? and (e) how can the mechanisms of change between outcome and process evaluation be elucidated in order to better understand the observed effects? In contrast to previous studies [29, 48], the selected ToC outcomes were aligned with appropriate indicators and guided the selection of suitable evaluation measures for the pilot test of the MENTUPP intervention. The indicators, measures, and results of the MENTUPP pilot study will be reported in a different publication.

Another strength of the developed ToC is that there is an explicit focus on the assumptions underlying the intervention across different implementation contexts, something that has not been reported in the context of previous ToC literature [29]. The workshops mentioned in the "Development steps of the ToC" section aimed at defining assumptions in an understandable manner that could be applied across all work sectors and countries. Consideration of factors that could potentially affect the efficacy and effectiveness of the intervention at the individual, organizational and national levels was also given.

An additional strength was that the theoretical framework we developed for the evaluation of the complex MENTUPP intervention integrated outcomes that are evidence-based in accordance with recommendations by the MRC framework [27] as they derive from the multiple reviews and an expert consultation conducted within MENTUPP. The selected evidence-based outcomes have been further utilized by the core research team and those involved in the ToC workshops to explain how and why one outcome is expected to lead to another and how the MENTUPP ToC intervention components will contribute to this end. The development of the ToC shares key characteristics with pre-existing interventions, although our approach also allows for the opportunity to elaborate on the causal mechanisms within the intervention and test hypotheses, taking into consideration its context and complexity. This detailed and evidence-informed approach can therefore be replicated in future studies.

The participatory process we followed to develop the proposed ToC for MENTUPP is an additional strength. Contrarywise to previous research [49, 50], a large number of partners representing all relevant disciplines, committed themselves to developing the MENTUPP ToC and made significant contributions to all development stages.

Preparing a ToC approach in accordance with guidelines based on the MRC framework, facilitated focusing on the role of the contextual complexity behind the intervention. The contextual characteristics derived from the social, political, economic, and organizational background may be used to predict and explain the adoption, effectiveness, and maintenance of the intervention. Contextual complexity is furthered by SMEs from three work sectors participating across the nine partner countries. The ToC will therefore be simultaneously tested in these different country contexts. Additionally, the developed theory will be tested via the MENTUPP pilot and will inform the broader cRCT study. Hence, there is an opportunity to test the use of a ToC approach with the MRC framework within a clustered RCT, meeting two under-explored fields in the literature [30].

Nevertheless, it is important to acknowledge some limitations. First, despite the reviews and the expert consultation conducted within the MENTUPP consortium to identify the evidence-base of workplace mental health interventions, the evidence identified remains limited, with evidence for interventions implemented specifically in SMEs being especially sparse. This lack of existing evidence led the MENTUPP intervention components to be largely developed by the participants of the ToC workshops. This also meant that components were limited to evidence that was considered appropriate specifically for the MENTUPP intervention. Nonetheless, this study will contribute towards addressing a key gap in the literature by generating shared robust research concerning the implementation of mental health interventions in SMEs and workplace settings in general. For this purpose, the developed ToC will be further optimized at a later stage using the pilot results and experience and will be able to gain a deeper understanding of the barriers and facilitators to implementation and effectiveness in a SME context.

Second, there is a risk that a comprehensive ToC map may be too detailed and complicated. Integrating all the proximate and intermediate outcomes required for the long-term outcomes would lead to an extended list of indicators and a very complex ToC map. To overcome these difficulties, we categorised all outcomes involved in MENTUPP in thematic groups necessary to evaluate the key elements and steps of the project. Although a limitation, this could also be considered an advantage as a ToC can be used as a background to focus more on specific parts of a study according to the main interest of the researchers. This means that it can be used to develop a more detailed ToC based on a wider one.

Third, the development process involved members of the MENTUPP consortium who had extensive knowledge of the intervention and could significantly contribute to the description of the assumed mechanisms underlying the intervention. However, there could have been benefits of also involving people affiliated with SMEs who could bring added value and integrate into our ToC the insider’s perspective on how the intervention could work in their organizations.

Fourth, the 6-month duration of the MENTUPP pilot was conducted during the COVID-19 pandemic which had a strong influence on all work sectors and was not considered long enough to achieve structural workplace changes such as adapting the level of job demands or increasing employee control. Therefore, the main focus of the intervention regarding changes to the organizational level is mostly related to the improvement of social support as a preliminary and required step for future structural improvement. Moreover, enhancing the ability to screen for psychosocial stressors in the workplace is one of the outcomes indicated by the intervention and the ToC. More structural factors can be targeted in the cRCT stage. Nevertheless, the developed ToC is considered to be a valuable source to guide the evaluation of future projects on how to integrate and link multiple outcomes making explicit the assumed connection between their causal mechanisms.

Fifth, it is important to acknowledge that innovative research is accompanied by the limitation that the results have not been verified through replication. Nonetheless, the proposed ToC will strengthen the systematic evaluation and verification of hypotheses related to the anticipated impacts of the MENTUPP intervention which is an ideal research opportunity for generalizability when it comes to the reproduction of its theoretical model for evaluation in future projects.

## Conclusions

Within this study, we developed a ToC model that illustrates a theory of how a complex mental health intervention is expected to work in order to achieve the desired long-term outcomes. The intervention components for the workplace-based mental health intervention are explicitly stated, while contextual factors and individual characteristics that can facilitate implementation, efficacy and effectiveness are also highlighted. Research has shown that there is an unmet need for mental health interventions following an integrated approach in the workplace and this ToC provides a first comprehensive model of how such interventions can be designed and evaluated. This ToC has the potential to inform and optimize the development, implementation, and evaluation of future work-related mental health promotion projects of high complexity.

## Supplementary Information

**Additional file 1.**

## Data Availability

The data derive from recordings of our workshops that have been transcribed and there are available reports.However, these data are very sensible and we cannot share them at the moment as they have to remain confidential by the end of the MENTUPP project which is located in 2024. Nonetheless, there is a detailed overview of the purpose and the outputs of each one of the workshops included in the manuscript. When the project will be completed, all of our data will be saved together in an open access repository. The BMC journal will be informed about the name of the repository as soon as it will be available. To request the data you can get in contact with Fotini Tsantila (fotini.tsantila@kuleuven.be), dr. Evelien Coppens (evelien.coppens@kuleuven.be) and Prof. dr. Chantal Van Audenhove (chantal.vanaudenhove@kuleuven.be).

## Abbreviations

ToC: Theory of Change
WHO: World Health Organization
SMEs: Small and Medium Enterprises
MENTUPP: Mental Health Promotion and Intervention in Occupational Settings
cRCT: Clustered randomized controlled trial
GDP: Gross domestic product
MRC: Medical Research Council framework
IGLO: Individual -Group-Leader-Organizational levels
CPO: Context-Process-Outcomes evaluation model
POs: Proximate Outcomes
IOs: Intermediate Outcomes
LOs: Long-term outcomes
CBT: Cognitive Behavioral Therapy
