# Supplementary material for: Developing a framework for evaluation: a Theory of Change for complex workplace mental health interventions
Source: BMC Public Health. 2023 Jun 17;23:1171. doi: 10.1186/s12889-023-16092-x (PMC10276374; doi:10.1186/s12889-023-16092-x)
Supplement: Supplementary file 1 — Additional file 1. [file 12889_2023_16092_MOESM1_ESM.docx]

**Table S1:** Overview of the MENTUPP rationales.

| Rationales | Evidence source | Link to ToC |
| --- | --- | --- |
| 1. Mental health interventions for the workplace should target mental wellbeing, stress, depression and anxiety and substance use disorders. | (52-54) | Reasoning behind the selection of the LOs. |
| 1. Creating a positive working environment could prevent mental health problems and reduce productivity losses in the workplace. | (55-58) | Reasoning behind the assumed relationship between productivity losses and the rest LOs. |
| 1. MENTUPP has to be perceived as useful so people will be persuaded to participate and promote it. | (59) | Reasoning behind assumption No4. |
| 1. The MENTUPP Hub has to be user-friendly and sector-appropriate to be perceived as useful. | (60) | Reasoning behind the creation of sector-specific components and assumptions No5 and 6. |
| 1. The implementation process and the engagement to MENTUPP can be promoted through the selection of the SME champion and through implementation processes enhancing participant’s capacity. | (61-63) | Reasoning behind PO1 and assumption No2. |
| 1. Supervisors do not only bring changes at the organizational level. They should be perceived as persons with mental health needs who can also benefit from interventions targeting individuals. | (44) | Reasoning behind including leaders in the outcomes assumed to be achieved by all employess (blue boxes in ToC map), but also connecting leaders to outcomes expected by them due to their role in the organizations (yellow boxes in ToC map). |
| 1. A multilevel approach combining interventions at the individual and the organizational level is recommended in order to achieve effectiveness. | (64) | Reasoning forintegrating components and therefore expected outcomes on the organizational level in the MENTUPP ToC (green boxes in ToC map). |
| 1. Stress, burnout and depressive symptoms can be prevented by managing psychosocial factors in the work environment. | (21, 65, 66) | Reasoning behind the assumed relationship between IO1 and LOs1-4. Thoroughly described in domain “Improving psychosial work factors” of section 3.5 in this article. |
| 1. Developing positive mental wellbeing is needed for everyone: for people who do and do not suffer from mental illness. | (22) | Reasoning behind introducing the intervention to all the employees in the participating organizations. |
| 1. Mental health and mental wellbeing are promoted by enhancing positive aspects and reducing work-related risk factors. | (21, 38) | The MENTUPP intervention components have been created in order to apply an integrated approach to workplace mental health through (i) protecting mental health by reducing work-related and other risk factors for mental health problems (PO6,IO1), (ii) promoting mental health by developing the positive aspects of work as well as worker strengths and positive capacities (IOs2-3, PO3) , and (iii) responding to mental health problems as they manifest at work regardless of cause (work-related or otherwise) (PO5) (14). |
| 1. CBT-based interventions reduce symptoms of depression and anxiety in workplaces. | (24, 36, 37) | Reasoning behind the integration of elements that are considered essential to any CBT mental health intervention (e.g., focus on pleasant activities, identification of cognitive distortions, and development realistic counterthoughts, mood monitoring, and relaxation and breathing techniques) (PO2-4). |
| 1. CBT-based interventions can be implemented on an affordable and wide scale (e.g. in group settings or by providing educational material). | (24, 37-39) | Additional reasosing to integrate CBT-based intervention components in a totally online intervention also considering assumption No2 about the limited resources in SMEs. |
| 1. Psychoeducational materials, face-to-face workshops and interventions based on CBT are rated as most useful and acceptable. | (67) | In addition to the reasoning about CBT-based interventions, psychoeducational intervention components providing information about wellbeing, mental illness, and the related stigma have been integrated in the MENTUPP Hub platform as described in domains “Building knowledge” and “Enhancing skills” in section 3.5 of this article. Face-to-face workshops were not able to take place due to COVID-19, but scenarios and videos describing possible cases of mental illness were included in the intevention components (PO2-4). |
| 1. Prevention, early recognition and treatment of anxiety and depressive disorders are crucial aspects of workplace interventions. | (68) | Reasoning behind components to enhance the mental health literacy of employees and leaders (PO2, PO5). |
| 1. Focusing on return to work in workplace-based interventions results in a quicker return to work. | (69) | Reasoning of including information about returning to work after sick-absense due to mental illness (PO2, PO5) and investing on the creation of a more inclusive atmosphere in the workplace (IO2-3). |
| 1. People facing depressive symptoms at a clinical level should be provided with additional help to overcome their difficulties and achieve change. | (15) | Reasoning behind the inclusion of the components described in domain “Arranging additional support” of section 3.5 in the article. |
| 1. Stigmatizing attitudes toward mental illness and help-seeking are important barriers for people with mental health problems to obtain adequate professional help. | (41, 42) | Reasoning behind components desrcibed in domain “ Adopting more positive attitudes” of sector 3.5 in the article and link to PO4 and IO4. |
| 1. Employees often hide their mental health difficulties due to stigma, hence appropriate anti-stigma programs are needed. | (67) | An additional reasoning to include the combat of stigma in MENTUPP. |
| 1. Self-organized peer support could be helpful in preventing and dealing with mental health difficulties. | (70) | Reasoning of perceiving peer-support as an important outcome to be achieved through MENTUPP (IO2) which is linked to better psychosocial factors (IO1, IO3) and to the LOs of MENTUPP. |
| 1. Enhanced Knowledge, skills and attitudes towards mental health can lead to behavioural change, if put into practice. | (33, 40) | Reasoning behind the assumed relationship between the POs and the IOs in the ToC map while taking into consideration the assumptions No3,7,9 which will facilitate the implementation. |
| 1. The majority of the employees in an SME should be involved in the intervention in order to achieve change. | (43) | An additional reasoning to involve all the employees. |
